# AI-driven preclinical disease risk assessment using imaging in UK biobank

**DOI:** 10.1038/s41746-025-01771-3

**Published:** 2025-07-26

**Authors:** Dmitrii Seletkov, Sophie Starck, Tamara T. Mueller, Yundi Zhang, Lisa Steinhelfer, Daniel Rueckert, Rickmer Braren

**Affiliations:** 1 Institute of Diagnostic and Interventional Radiology, Technical University of Munich School of Medicine, Munich, Germany; 2 Chair for AI in Healthcare and Medicine, Technical University of Munich (TUM) and TUM University Hospital, Munich, Germany; 3 Department of Computing, Imperial College London, London, UK; 4 German Cancer Consortium (DKTK), Munich partner site, Heidelberg, Germany

**Keywords:** Diseases, Risk factors

## Abstract

Identifying disease risk and detecting disease before clinical symptoms appear are essential for early intervention and improving patient outcomes. In this context, the integration of medical imaging in a clinical workflow offers a unique advantage by capturing detailed structural and functional information. Unlike non-image data, such as lifestyle, sociodemographic, or prior medical conditions, which often rely on self-reported information susceptible to recall biases and subjective perceptions, imaging offers more objective and reliable insights. Although the use of medical imaging in artificial intelligence (AI)-driven risk assessment is growing, its full potential remains underutilized. In this work, we demonstrate how imaging can be integrated into routine screening workflows, in particular by taking advantage of neck-to-knee whole-body magnetic resonance imaging (MRI) data available in the large prospective study UK Biobank. Our analysis focuses on three-year risk assessment for a broad spectrum of diseases, including cardiovascular, digestive, metabolic, inflammatory, degenerative, and oncologic conditions. We evaluate AI-based pipelines for processing whole-body MRI and demonstrate that using image-derived radiomics features provides the best prediction performance, interpretability, and integration capability with non-image data.

## Introduction

Risk assessment and stratification aim at early identification of individuals before disease onset or at a preclinical disease stage, enabling primary prevention and timely intervention. This proactive approach has the potential to shift healthcare from treatment to anticipation and prevention, reducing the burden of advanced disease and improving long-term outcomes^1,2^. At the same time, with advances in medical imaging technologies, there is a growing interest in integrating image data into the prediction and detection process. The advent of artificial intelligence (AI) has further revolutionized this field, enabling the detection of subtle changes and the extraction of complex patterns and features that are beyond human perception^3,4^. Combining imaging with other clinical and lifestyle information has already demonstrated benefits in diagnostic prediction^5–7^ and, therefore, represents a promising direction for improving the accuracy in a prognostic risk assessment task.

In recent years, numerous approaches have leveraged non-image data for risk assessment in various diseases. Dolezalova et al. develop tools for predicting cardiovascular disease (CVD)^8^ and Type 2 Diabetes (T2D)^9^ risk based on features that can be collected outside of a clinical setting, without requiring specialized medical equipment or an in-person visit to a healthcare provider. In contrast, Steinfeldt et al.^10^ utilize clinical information to predict the 10-year risk of major CVD events. Expanding on these efforts, Mamouei et al.^11^ investigate the impact of a range of factors on CVD risk, including medical events, behavioral and socioeconomic influences, environmental conditions, and clinical measurements. Beyond CVD, other conditions have also been explored. Julkunen et al.^12^ evaluate the prognostic value of metabolic blood biomarkers in CKD. Meng et al.^13^ evaluate risk factors for alpha-1 antitrypsin deficiency-associated liver disease (AATD-LD), including disease characteristics, laboratory values, demographics, and lifestyle factors, to predict clinical outcomes such as all-cause mortality, liver-related death, and likelihood of liver transplant. Shifting the focus to a broader spectrum of age-related diseases, Lian et al.^14^ examine metabolic biomarkers and Gadd et al.^15^ blood protein levels as key indicators of disease risk and mortality. Cancer risk assessment with non-image data has been approached from multiple angles as well. Sun et al.^16^ analyze liver function markers for lung cancer, while for general cancer, Soto et al.^17^ and Chang et al.^18^ investigate dietary factors, such as diet type and ultra-processed food, respectively. Placido et al.^19^ focus on integrating disease history into pancreatic cancer risk prediction models.

Image-based approaches are less frequently used in the context of risk assessment. Flynn et al.^20^ use DXA knee scans to extract the minimum joint space width (mJSW) feature, which is then linked with genetic data to derive a polygenic risk score (PRS) for knee osteoarthritis. Huang et al.^21^ derive features from abdominal ultrasonography, carotid artery ultrasonography, bone mineral density scans, and electrocardiography to improve T2D risk assessment based on genetic data. Prasad et al.^22^ utilize retinal fundus images to analyze vascular patterns for CVD prediction. In breast cancer risk assessment, Eriksson et al.^23^ extract mammographic features such as density, microcalcifications, masses, and asymmetries from full-field digital mammography. Additionally, Linge et al.^24^ examine muscle composition and liver features extracted from whole-body and liver MRIs for their predictive value on all-cause mortality. For non-small cell lung cancer (NSCLS) risk stratification, Vanguri et al.^25^ and Captier et al.^26^ employ multi-modal approaches that integrate radiomics features extracted from computed tomography (CT) images with the clinical and pathological data. Despite the growing use of imaging in disease risk assessment, the application of radiation-free whole-body MR screening exams remains an underexplored area, particularly in understanding how they can be effectively incorporated into screening and prediction algorithms. This integration could significantly improve the detection of preclinical disease stages and personalized risk stratification across a wide range of disease conditions due to its ability to provide an objective and comprehensive, multi-organ view in a single exam.

In this work, we utilize various AI models to explore low-resolution whole-body MR images, thereof-derived radiomics features, and non-image data for a 3-year preclinical risk assessment of CVD, pancreatic disease, liver disease, cancer, COPD, CKD, and osteoarthritis. We additionally investigate the potential of functional and structural image-derived cardiac features extracted from higher-resolution cardiac MRI for CVD risk assessment. Our findings demonstrate that images and image-derived features are powerful predictors of preclinical disease risk, enhancing the predictive value of non-image data. We also show that in terms of risk prediction accuracy, employing image-derived whole-body radiomics and cardiac features outperforms the direct use of whole-body MRI. These features provide better interpretability and training efficiency while being well-suited for integration with non-image tabular data, enabling more effective multi-modal analysis.

## Results

### Preclinical risk assessment in UK biobank

We use UK Biobank^27^ to identify the disease groups - CVD, pancreatic disease, liver disease, cancer, COPD, CKD, and osteoarthritis - using a disease-specific set of International Classification of Diseases (ICD-10) codes and self-reported information as filtering criteria. An event is defined as the recorded occurrence of a disease-specific diagnosis in the linked health sources, such as a cancer register, hospital records, and self-reported information. Subjects are classified as at-risk for a particular disease if their first event occurs within three years after the imaging assessment and does not occur before or within three months; otherwise, if no event is ever recorded, they are classified as healthy. We extract an equal number of at-risk and healthy subjects with aligned distributions of age, sex, body mass index (BMI), and ethnicity within the disease-specific dataset by applying propensity score matching^28^. The resulting balanced dataset is randomly split into training, validation, and test sets. More details on the disease group identification and training regime are provided in the Methods Section.

**Fig. 1:**
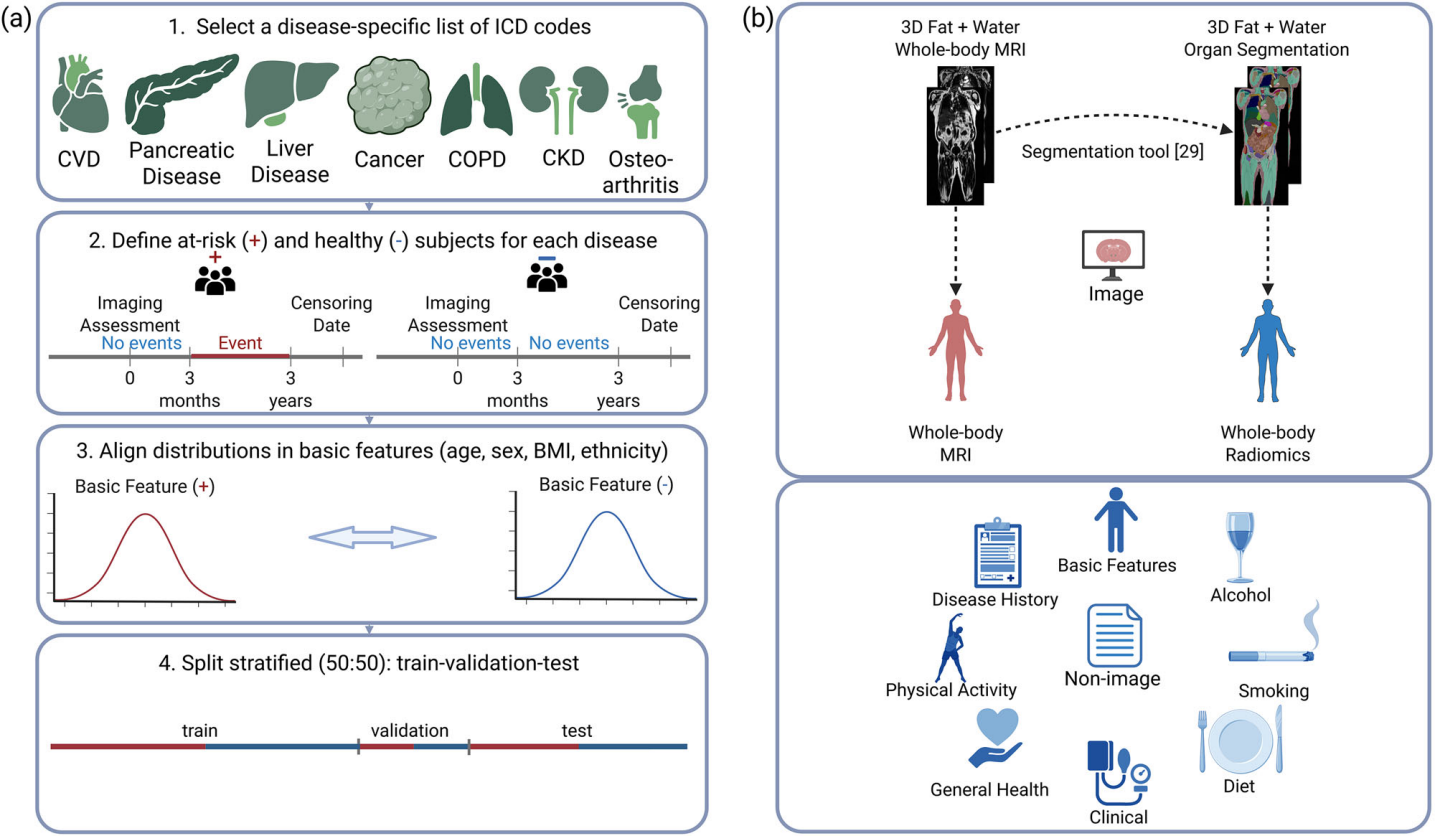
Dataset construction for preclinical disease risk assessment in UK Biobank, including. **a** Selection pipeline using ICD-10 codes and self-reported information from the linked health sources, and (**b**) collecting multi-modal data, whole-body MRI (upper and red), extracted whole-body radiomics (upper and blue), and non-image data (lower and blue). Created in BioRender. Seletkov, D. (2025) https://BioRender.com/x14itf2.

To capture a subject’s profile, we integrate multiple data modalities. As a non-image modality, we incorporate general information related to lifestyle, sociodemographics, and health. We identify the features from the following categories available in the UK Biobank: basic features, clinical features, disease history before imaging assessment, physical activity, general health features, diet, smoking, and alcohol habits. As the imaging modality, we select the 3D whole-body MRI with fat and water contrasts. We apply a whole-body MRI segmentation tool^29^ to segment 69 different organs and extract whole-body radiomics features. For CVD, we employ additional cardiac structural and functional features extracted from the cardiac MRI, as described by Bai et al.^30^. The overview of dataset construction is shown in Fig. 1 and the resulting disease-specific datasets in Table 1.

**Table 1:** Overview of resulting preclinical disease risk datasets using UK Biobank with a number of subjects in subsets and average characteristics of at-risk and healthy groups

		# of subjects in subsets			Average statistics			
Dataset	Group	Train	Validation	Test	Age	BMI	% Female	Time-to-event [days]
CVD	at-risk	570	64	159	66.3 ± 7.4	27.0 ± 4.5	44.4%	574.6 ± 279.3
	healthy	570	63	158	66.3 ± 6.9	25.9 ± 4.0	44.1%	no event
Pancreatic Disease	at-risk	212	24	59	65.8 ± 7.4	29.6 ± 5.8	35.6%	528.3 ± 277.1
	healthy	212	24	59	65.7 ± 6.6	26.2 ± 3.7	33.9%	no event
Liver Disease	at-risk	146	17	41	64.5 ± 8.2	28.9 ± 5.6	50.7%	592.5 ± 290.4
	healthy	145	16	41	64.2 ± 8.0	26.8 ± 4.2	50.2%	no event
Cancer	at-risk	432	49	121	65.9 ± 6.9	27.1 ± 4.5	40.0%	492.9 ± 268.9
	healthy	432	48	120	65.8 ± 6.9	26.5 ± 4.1	40.6%	no event
COPD	at-risk	139	16	39	67.9 ± 7.2	27.5 ± 4.9	39.9%	529.5 ± 278.5
	healthy	138	15	39	68.1 ± 7.1	26.6 ± 4.4	40.6%	no event
CKD	at-risk	219	25	61	69.9 ± 6.2	28.1 ± 4.8	45.9%	561.7 ± 286.4
	healthy	218	24	61	69.7 ± 6.2	26.8 ± 4.3	44.4%	no event
Osteoarthritis	at-risk	662	74	184	66.5 ± 7.0	27.3 ± 4.6	54.1%	567.8 ± 274.7
	healthy	662	74	184	66.5 ± 6.9	26.4 ± 4.2	54.8%	no event

Due to the distinct nature and dimensionality of investigated data modalities, such as whole-body MRI and tabular data encompassing non-image and image-derived whole-body radiomics and cardiac features, we employ specialized AI models^31,32^. Specifically, ResNet18 3D^33^ is trained on the whole-body MRI, while Random Forest (RF)^34^, eXtreme Gradient Boosting (XGB)^35^, and Multi-Layer Perceptron (MLP) on non-image and image-derived whole-body radiomics and cardiac features. To investigate multi-modal performance, we combine tabular image-derived and non-image features at the input level. For whole-body MRI, we apply joint and late fusion strategies^5^ to combine the images with non-image features and report the best results achieved.

**Fig. 2:**
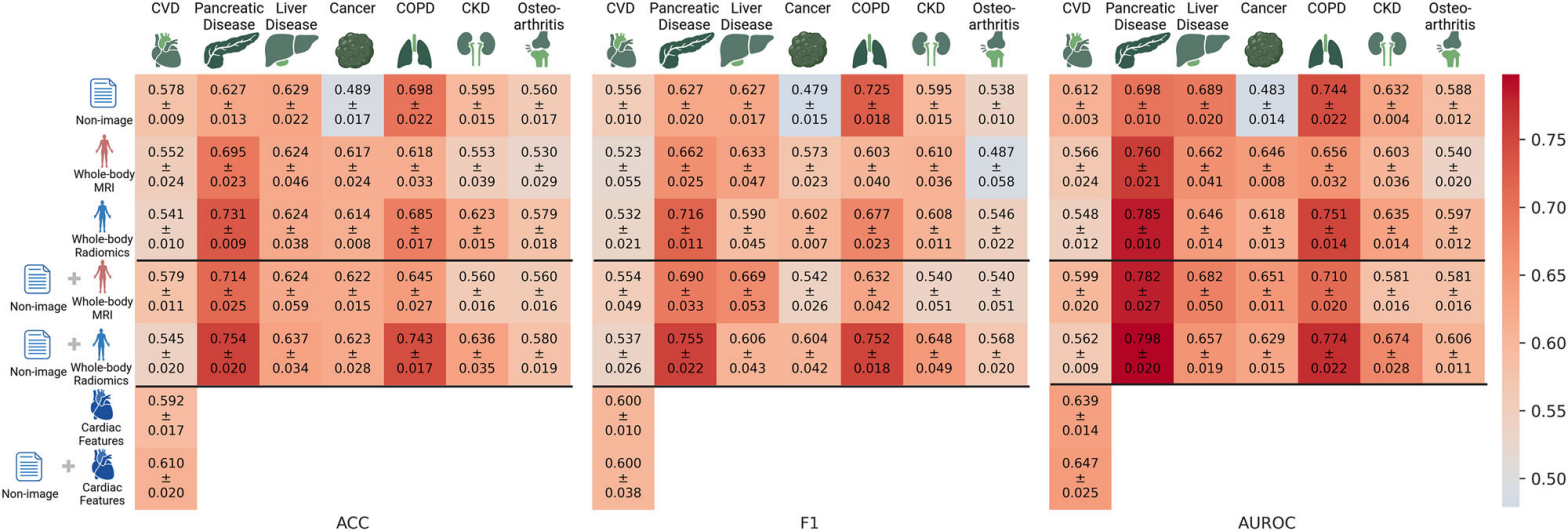
Results of 3-year preclinical risk assessment for cardiovascular disease (CVD), pancreatic disease, liver disease, cancer, chronic obstructive pulmonary disease (COPD), chronic kidney disease (CKD), osteoarthritis. The rows represent the data modality, in order: non-image, whole-body MRI, whole-body radiomics extracted from whole-body MRI, non-image with whole-body MRI, non-image with whole-body radiomics, cardiac features^30^, and non-image with cardiac features. Created in BioRender. Seletkov, D. (2025) https://BioRender.com/m09n1fh.

Building these multi-modal disease-specific datasets and modality-specific AI models, we investigate the 3-year preclinical risk assessment as a binary classification problem. All models are trained to optimize accuracy (ACC), the primary evaluation metric. We additionally report the F1 score (F1) and area under the receiver-operating characteristic curve (AUROC) for completeness. The results of the best-performing models across all modalities and datasets are presented in Fig. 2.

### Unimodal analysis

To understand the impact of each modality, we first explore the non-image data, whole-body MRI, and whole-body radiomics features individually.

The non-image features are predictive for the risk assessment of all diseases except cancer. We assume that this is due to a broad definition of cancer in our study, which includes malignancies originating from various primary organs, and a strong genetic component in cancer development^36^.

Whole-body MRI achieves a mean accuracy above 0.61 for pancreatic disease, liver disease, cancer, and COPD, while close-to-random performance is observed for CVD, CKD, and osteoarthritis. However, the whole-body radiomics features perform better in cases of pancreatic disease, COPD, CKD, and osteoarthritis and on par with CVD, liver disease, or cancer. We hypothesize this with the ability of radiomics features to extract consolidated patterns, reducing the noise and irrelevant information inherent in whole-body MRI. Additionally, the tabular models used with radiomics undergo rigorous hyperparameter tuning, optimizing their predictive performance. In contrast, the higher-dimensional whole-body MRI data poses greater challenges for effective training, potentially limiting its performance for certain datasets.

To investigate the effects of varying dataset sizes, shown in Table 1, and the use of high-parameterized ResNet18 3D, we additionally evaluate a custom CNN model with fewer trainable parameters in Supplementary Fig. 1. Findings show that ResNet18 3D performs comparably or better, validating its selection for consistency in subsequent analyses.

We investigate the poor performance of the imaging modalities for CVD. We hypothesize that the information related to the accurate CVD risk assessment may be present in the image but cannot be fully captured by static low-resolution whole-body MRI. To address this, we evaluate cardiac functional and structural features extracted from higher-resolution cardiac MRI. We observe that this imaging modality contains more relevant information, making it better suited for CVD risk assessment. We additionally evaluate the cardiac features in combination with whole-body radiomics for other datasets and conclude that the cardiac features also improve the performance for liver disease risk assessment, consistent with the known link between cardiovascular, metabolic, and hepatic pathophysiology^37^. The detailed results are reported in Supplementary Fig. 2.

### Multi-modal analysis

We explore the fusion of non-image data with whole-body MRI, whole-body radiomics, and cardiac features. The integration of non-image data with whole-body radiomics shows better performance compared to whole-body MRI in terms of accuracy across all datasets except CVD, where the fusion with image data does not significantly improve the non-image baseline. Notably, the fusion with whole-body MRI excels for liver disease and cancer in terms of AUROC, consistent with unimodal experiments. The fusion performance of whole-body radiomics and whole-body MRI is attributed to several factors. First, whole-body radiomics features demonstrate comparable or superior performance to whole-body MRI in unimodal settings. Second, the fusion of image and tabular data can be achieved through late or joint fusion^5^. However, late fusion can be suboptimal due to a lack of interactions between features from different modalities^5,7^, and despite being end-to-end trainable, joint fusion using MLP models is less effective than not end-to-end trainable tree-based algorithms such as XGB or RF, which yield the best results, shown in Supplementary Tables 1–7. Furthermore, tabular models undergo extensive hyperparameter tuning, allowing iteration over thousands of hyperparameters due to their short training times in seconds compared to the days required for image models, making the hyperparameter tuning process infeasible.

As shown in Supplementary Fig. 3 and corresponding Supplementary Tables 8–14, late fusion surpasses joint fusion in most experiments, with a minimal performance gap in those where joint fusion excels. This supports adopting late fusion, which offers practical benefits, including combining unimodal models without additional training and flexibility in model substitution. Additionally, we investigate the potential influence of dataset size on fusion strategy performance for the “non-image + whole-body MRI” experiment in Supplementary Fig. 4. No consistent pattern is observed. Among larger datasets with more than 1000 total samples, such as CVD, osteoarthritis, and cancer, both fusion methods show mixed results - late fusion performs better for CVD and Osteoarthritis, while joint fusion has slightly better performance for Cancer. Similarly, for the datasets with fewer samples, such as COPD, liver disease, pancreatic disease, and CKD, the minimal and inconsistent performance differences are observed. These findings suggest that dataset size does not systematically favor one fusion strategy over the other.

Except for CVD, where whole-body MRI and radiomics features show limited predictive capability, our findings demonstrate that fusing non-image data with whole-body radiomics features consistently outperforms individual modalities in terms of average accuracy. This fusion approach also exhibits the best performance across all datasets. Similarly, for CVD, the fusion of non-image data with cardiac features indicates superior performance compared to other experiments.

To assess the generalization performance and feature importances of our models, we employ nested cross-validation with non-overlapping stratified 5 outer and 10 inner folds across 5 seeds, mimicking the initial fixed train-validation-test evaluation that allowed us a fair comparison with the “whole-body MRI” experiment. The results are reported in Supplementary Fig. 5 and do not exhibit a significant difference in performance evaluation.

Cross-validation allows us to investigate the importance of the different feature categories across diseases using the best-performance models for the “non-image + whole-body radiomics” experiment applied to the test datasets in each outer fold. We chose the “non-image + whole-body radiomics” model for feature importance analysis since it consistently achieves the best predictive performance across datasets and enables us to explore the added value of image-derived features when combined with non-image information, offering a more comprehensive view of the most informative organ systems.

We compute the mean feature importance across all test datasets for each category using the model-independent permutation importance method^34^ with 100 shuffles. Only the statistically significant feature categories are used (*p*-value < 0.05*14 Bonferonni corrected for non-image and 13 whole-body radiomics categories in each dataset).

**Fig. 3:**
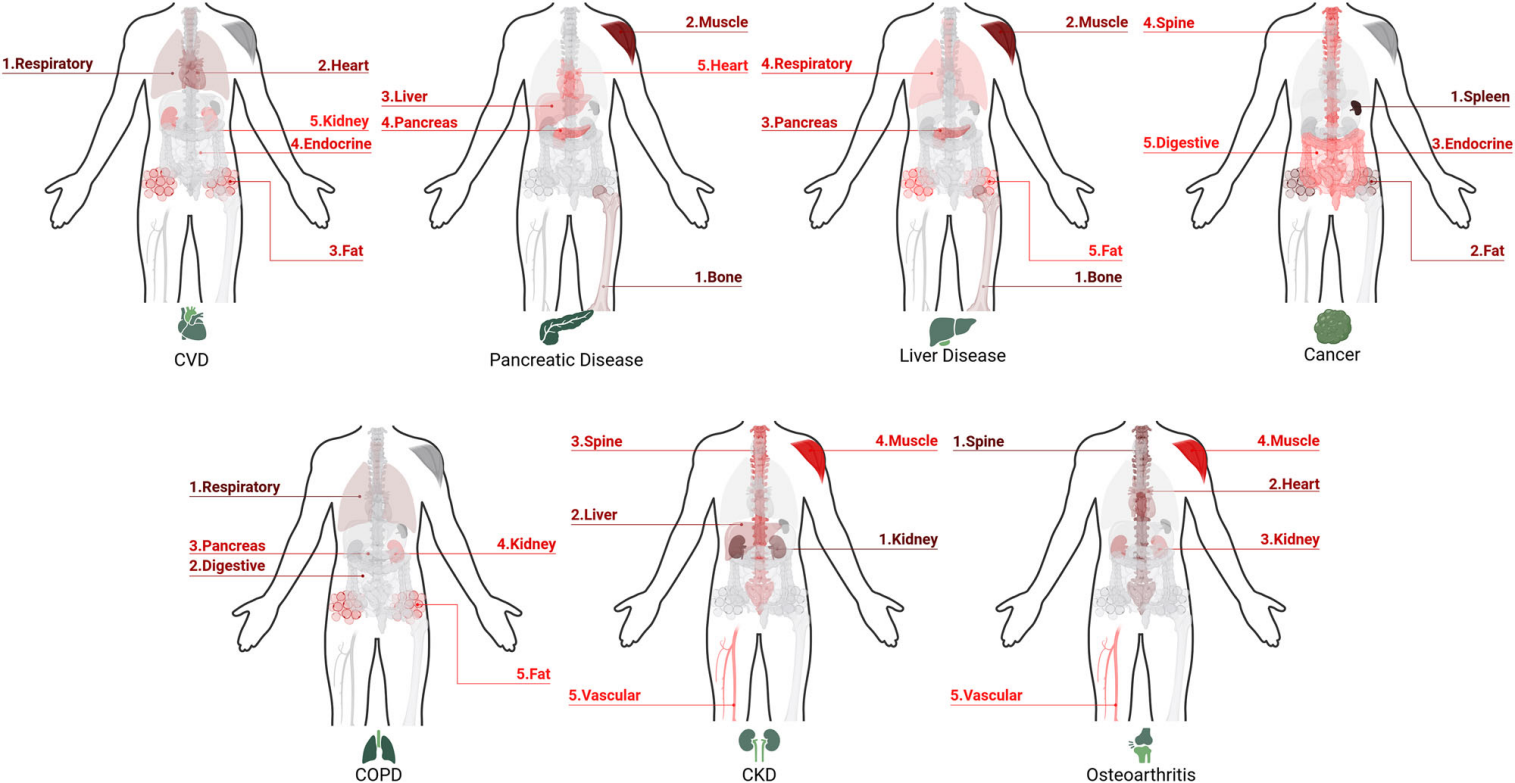
Top-5 organ systems by feature importances for 3-year preclinical risk assessment for cardiovascular disease (CVD), pancreatic disease, liver disease, cancer, chronic obstructive pulmonary disease (COPD), chronic kidney disease (CKD), osteoarthritis in the “non-image + whole-body radiomics” experiment. The feature importances are calculated for 13 organ systems: heart, vascular, respiratory, digestive, liver, pancreas, spleen, endocrine, kidney, spine, bone, muscle, and fat. Created in BioRender. Seletkov, D. (2025) https://BioRender.com/n21d793.

Figure 3 illustrates the five most important organ systems by feature importance ranking for each disease. The absolute feature importances of all categories across all diseases are reported in Supplementary Fig. 6.

We observe that in addition to organ system-specific categories linked to particular diseases - such as respiratory for COPD, kidney for CKD, and spine for osteoarthritis - the features related to bone, fat, and muscle also hold high importance in risk prediction across various diseases, particularly pancreatic and liver diseases. This finding is expected, given the fundamental role of these organs in nutrition and metabolism.

We additionally provide the absolute feature importances for the “whole-body radiomics” experiment in Supplementary Fig. 7 and discuss in Supplementary Note 1.

## Discussion

In this work, we demonstrate how image data facilitates risk prediction for a wide range of diseases at an early time point, that is, in a preclinical disease stage, both as a standalone modality and in combination with non-image data. In particular, we highlight the potential of whole-body MRI, which offers a comprehensive multi-organ view in a single screening exam, as opposed to non-image questionnaire data, which often depends on self-reported information and is prone to bias and inaccuracies.

In our study, we apply modality-specific AI models to whole-body MRI and image-derived features from whole-body and cardiac MRI, and non-image questionnaire and clinical data from the UK Biobank. This enables an assessment of the 3-year preclinical risk of CVD, pancreatic disease, liver disease, cancer, COPD, CKD, and osteoarthritis.

Our findings demonstrate that image-derived features offer several advantages compared to images. The image-derived features are generally easier to handle, more interpretable, and exhibit superior performance in our experiments. This can be attributed to their condensed representation, which eliminates noise and irrelevant information often present in images. Specifically, we assume that the structured image-derived features allow the model to filter out artifacts, unrelated anatomical structures, or variations due to scanning parameters and patient positioning through segmentation and quantification pipelines. Furthermore, the lower dimensionality of image-derived features compared to images simplifies the extensive hyperparameter tuning, further enhancing model performance. As a result, image-derived features provide a more practical and efficient alternative to working directly with images.

The combination of image-derived whole-body radiomics or cardiac features with non-image data shows improved performance in risk prediction for most of the investigated diseases. These results highlight the complementary nature of each modality and their potential to offset individual limitations, paving the way for AI-driven, multi-modal preclinical disease risk assessment.

The results and generalizability of our experiments should be viewed under the following limitations. The analysis is based on self-reported and hospital in-patient data available in the UK Biobank. Our disease group selection strategy ensures only that the disease is not reported at the time of imaging assessment or within the following 3 months, and does not include the radiological validation of the MRI scans by medical experts. To maintain statistical validity, we are limited to broad disease categories in our experiments. Nevertheless, we believe that such an approach remains valuable and could trigger additional higher-resolution scans of certain organs or body regions in the clinical workflow.

The artificial 1:1 proportion of at-risk and healthy subjects in the training, validation, and test subsets is chosen intentionally to make the training on large whole-body 3D fat and water volumes computationally feasible. However, this choice restricts our ability to accurately assess real-world model performance, where the distribution of at-risk and healthy cases differs. Future work may prioritize assessing model performance under more realistic prevalence scenarios, including imbalanced test sets that better represent the true distribution of disease in the target population, where at-risk cases are typically less frequent. Furthermore, while we employ a 3-year risk assessment window, future studies could explore alternative time horizons, e.g., 4 and 5 years. In addition, an important direction for future work lies in developing more effective methods for integrating tabular and image data to enhance predictive performance.

An additional limitation of our radiomics pipeline is the reliance on the performance of the segmentation algorithm. Errors or biases in segmentation can propagate to downstream risk predictions. Previous work^38,39^ has shown that segmentation performance can vary across demographic groups, particularly when certain populations, e.g., by sex or ethnicity, are underrepresented in the training data. This may result in reduced accuracy or biased risk predictions in those subgroups and should be explored in future work.

We acknowledge that whole-body MRI is not currently used in routine screening due to high costs and the limited resolution, which lowers its sensitivity for disease detection. However, unlike CT, MRI offers a radiation-free approach, making it a safer option for large-scale population applications. Our findings provide proof of concept showing that whole-body MRI, even at lower resolutions, in combination with AI-driven image analysis, can capture meaningful information for preclinical risk assessment.

## Methods

### UK Biobank dataset

UK Biobank^27^ is a long-term population study following 500,000 volunteers 40–69 years of age at recruitment in 2006–2010. As a sub-study, 100,000 participants are recalled for a detailed imaging assessment, including a repeat of the baseline assessment. All assessments encompass a broad range of data, including sociodemographic, lifestyle, and linked health sources.

The imaging assessment includes, among other modalities, whole-body and cardiac MRIs. The whole-body MRI consists of a neck-to-knee T1-weighted dual-echo Dixon MR image with a size of [224 × 168 × 363] voxels and a resolution of [2.23 × 3 × 2.23] mm with water and fat contrasts. Cardiac MR (CMR) imaging consists of a multi-view 2D + Time image that comprises 2D slices from short-axis and long-axis views in the time dimension.

The UK Biobank has ethical approval from the North West Multi-centre Research Ethics Committee to handle human participant data, no additional ethical approval was required because the study involved the secondary use of data. Written informed consent was obtained from all participants and all data is deidentified for analysis. Eligible researchers may access UK Biobank data on www.ukbiobank.ac.uk upon registration. For this study, permission to access and analyze the UK Biobank data was approved under application 87802 from July 2022.

In our work, we define data as a collection of information, including health history, questionnaires, and imaging. Features are specific attributes extracted from data used for analysis. Biomarkers are the clinically relevant discovered features from biological processes.

### Construction of multi-modal risk assessment datasets from UK biobank

We identify the following disease groups, namely CVD, pancreatic disease, liver disease, cancer (excluding melanoma and other malignant neoplasms of skin), COPD, CKD, and osteoarthritis, using linked health sources in the UK Biobank, including hospital in-patient summary diagnoses (field IDs 41270 and 41280), cancer register (field IDs 40006 and 40005), self-reported cancer (field ID 20006) and disease (field IDs 20002 and 20008) fields. For each disease, a set of ICD-10 and self-reported codes is specified by a medical expert on the basis provided by refs. ^40,41^. A detailed list of ICD-10 codes and data fields is reported in Supplementary Data 1. We define an event as the recorded occurrence of a disease-specific diagnosis in any of the available linked health sources. The earliest date of the event is considered when conflicting dates across linked health sources occur. A subject is classified as at-risk if the first occurrence of any disease-specific event is recorded within three years after the imaging assessment in the hospital in-patient summary diagnosis, and no relevant event appears before or within three months after it in any linked health source. The three-month exclusion period was chosen to minimize the risk of imminent yet unreported diagnoses at the time of the imaging assessment. Importantly, the same subject can be included in the at-risk cohort for multiple diseases if they meet the criteria for more than one condition. A subject is classified as healthy if no recorded event from the disease-specific list exists at any time before or after the imaging assessment in any linked health source, and the imaging assessment occurred at least three years before the censoring date provided by UK Biobank. This results in a higher proportion of healthy subjects than disease-specific at-risk cases.

To address the imbalance between at-risk and healthy cohorts, we apply propensity score matching^28^ based on age, sex, BMI, and ethnicity. Propensity scores are estimated using logistic regression, which predicts the probability of belonging to the at-risk cohort. We perform 1:1 nearest-neighbor matching without replacement to ensure that each individual in the at-risk cohort is matched to an individual from the healthy cohort. This matching process provides a similar distribution of the variables age, sex, BMI, and ethnicity across both cohorts, minimizing their potential confounding effects. The resulting balanced dataset is randomly split into training (72%), validation (8%), and test (20%) sets, maintaining the balance between at-risk and healthy cases in each subset. Table 1 presents the number of subjects in each subset of the extracted datasets and demonstrates the results of the propensity score matching in aligning age, BMI, and sex. The results of the statistical testing and absolute standardized mean differences for the comparison of the at-risk and healthy groups in age, BMI, and sex are reported in Supplementary Tables 15 and 16, respectively. These demonstrate the achieved balance in age and sex variables between at-risk and healthy groups, but the difference in BMI, especially in pancreatic and liver diseases, indicating the limited pool of healthy subjects with sufficiently high BMI to match the corresponding at-risk cases, making the balance harder to achieve. The average time-to-event shows the average temporal interval between the imaging assessment and the first event for corresponding diseases and ranges from 492.9 to 592.5 days, supporting the datasets’ suitability for a 3-year preclinical risk assessment.

As the next step, we collect relevant data available in the UK Biobank for multi-modal analysis. To represent non-image modality, we use the previous works^8,9,11,13,17,18^ and medical expert input to identify the features from the following categories available in the UK Biobank at the imaging assessment: basic features, clinical features, disease history before imaging assessment, physical activity, general health features, diet, smoking, and alcohol habits. Basic features include age, sex, BMI, and ethnicity. Clinical features include waist circumference, systolic and diastolic blood pressure, and forced expiratory volume in 1 second. Disease history is retrieved as the one-hot vector of the Elixhauser Comorbidity Disease classes^40^ based on ICD-10 codes from the hospital in-patient summary diagnoses. The disease-specific history is absent for at-risk and healthy subjects in the respective dataset, indirectly validating the correct dataset construction pipeline. General health features encompass self-rated overall health rating, long-standing illness, disability or infirmity, falls in the last year, and weight change compared with one year ago. Detailed information on features, including physical activity, smoking, alcohol, and diet habits, along with their corresponding field IDs for all non-image feature categories introduced above, is provided in Supplementary Data 1.

To represent the imaging modality, we select the 3D whole-body MRI with fat and water contrasts, which captures all organ systems related to the investigated diseases. We further extract whole-body radiomics features, using the whole-body MRI segmentation tool^29^ and PyRadiomics^42^. The radiomics include first-order statistics, gray level co-occurrence matrix, gray level run length matrix, gray level size zone matrix, neighboring gray-tone difference matrix, gray level dependence matrix, and shape-based features. Except for shape-based features, we extract radiomics features for fat and water contrasts separately.

For the CVD dataset, we employ additional cardiac structural and functional features^30^. These features contain volumetric measurements of all four cardiac chambers, including end-systolic and end-diastolic volumes of the left and right ventricles and maximum and minimum volumes of the left and right atria. Functional features contain volumetric measurements, such as ejection fractions, stroke volumes for all chambers, and ventricular cardiac output. Structural measurements include the ventricular mass and the detailed assessment of myocardial wall thickness. The wall thickness is measured globally and across 16 segments according to the American Heart Association (AHA) model, providing a comprehensive map of myocardial thickness. The full list of image-derived features is provided in Supplementary Data 1.

### AI pipelines

The non-image and image-derived cardiac and whole-body radiomics features undergo the following preprocessing pipeline^43^. First, continuous and categorical features are identified. The continuous missing values are imputed using the mean and subsequently standardized. Categorical features are encoded using ordinal encoding when applicable; otherwise, one-hot encoding is used. Sparse categorical one-hot features (occurring in less than 1% of cases) are merged into one ‘other’ category. The combination of features from different categories occurs at the input level.

The whole-body radiomics features are split into anatomical categories: heart, vascular, respiratory, digestive, liver, pancreas, spleen, endocrine, kidney, spine, bone, muscle, and fat. For each category, we select 20 features based on the mRMR^44^ feature selection algorithm, which maximizes relevance to the target variable while minimizing redundancy among features. Following the approach of Borga et al.^45^ for fat normalization, the shape features from bone, spine, muscle, and fat categories are normalized by height squared to account for individual body size differences. For all continuous features, including radiomics, the standard scaler is fitted to the training set and applied to validation and test.

Three distinct tabular models are trained: Random Forest (RF)^34^, eXtreme Gradient Boosting (XGB)^35^, and Multi-Layer Perceptron (MLP)^46^ using Scikit-learn^47^. Each model is trained for each target disease five times with different random seeds. Other hyperparameters are tuned using Tree-Structured Parzen Estimator in Optuna^48^ with 500 trials for each model and seed to optimize model accuracy. The hyperparameter space is limited to avoid overfitting and is reported in Supplementary Table 17.

For image data, ResNet18 3D^33^, a well-established model in the medical domain^49–51^, is trained using PyTorch^52^ on whole-body MRI. Fat and water contrasts are provided as two input channels. A separate model is trained for each target disease. The hyperparameters for the training are reported in Supplementary Table 18.

To merge tabular and image data, we experiment with both late and joint fusion methods^5^. Late fusion is performed by averaging the prediction probabilities from the previously described tabular and image models, requiring no additional training.

For joint fusion, an MLP encoder is utilized for the tabular data, while the same ResNet18 3D, modified by removing all fully connected layers to serve as an encoder, is employed for the image data. The image encoder is initialized with the weights from the image-only experiment. A fusion MLP is applied on top of image and tabular encoders, and the entire network is trained end-to-end. The latent dimensions of both the image and tabular encoders are set to be the same before fusion to facilitate the equal contribution of both modalities to the final prediction. Since tree-based models like RF and XGB cannot be jointly trained with CNNs, they are used only in late fusion.

## Supplementary information

Supplementary Information

Supplementary Data 1

## Data Availability

Eligible researchers may access UK Biobank data on www.ukbiobank.ac.uk upon registration. For this study, permission to access and analyze the UK Biobank data was approved under application 87802, with initial approval granted in July 2022.

## Code availability

The code used in this work is available at https://github.com/yayapa/ukbb_risk.
