# Supplementary material for: AI-driven preclinical disease risk assessment using imaging in UK biobank
Source: NPJ Digit Med. 2025 Jul 26;8:480. doi: 10.1038/s41746-025-01771-3 (PMC12297167; doi:10.1038/s41746-025-01771-3)
Supplement: Supplementary file 1 — Supplementary Information [file 41746_2025_1771_MOESM1_ESM.pdf]

# Supplementary Information

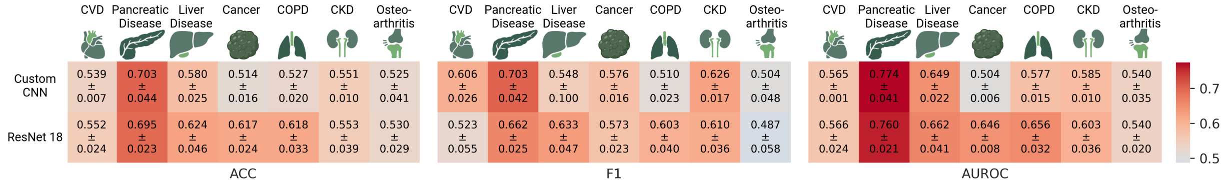


**Supplementary Figure 1.** Results of ResNet18 and a custom CNN model with fewer trainable parameters using whole-body MRI for 3-year preclinical risk assessment for cardiovascular disease (CVD), pancreatic disease, liver disease, cancer, chronic obstructive pulmonary disease (COPD), chronic kidney disease (CKD), osteoarthritis. The custom CNN consists of three 3D convolutional layers with kernel size 3, stride 1, and padding 1, each followed by batch normalization and $2 \times 2 \times 2$ max pooling. It ends with adaptive global average pooling and a fully connected layer for classification. Created in BioRender. Seletkov, D. (2025) https://BioRender.com/c7lrjbl.

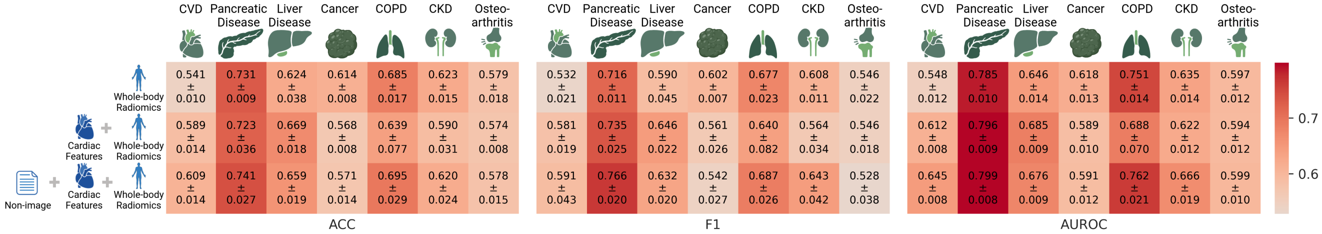


**Supplementary Figure 2.** Results of whole-body radiomics with cardiac features and non-image data fusion for 3-year preclinical risk assessment for cardiovascular disease (CVD), pancreatic disease, liver disease, cancer, chronic obstructive pulmonary disease (COPD), chronic kidney disease (CKD), osteoarthritis. The integration of cardiac features with whole-body radiomics results shows improved predictive accuracy for risk assessment of CVD and liver disease. A minor improvement in F1 and AUROC is observed by pancreatic disease. The observed findings demonstrate the known interplay between the cardiovascular, metabolic, and hepatic systems[1]. Created in BioRender. Seletkov, D. (2025) https://BioRender.com/fxchltj.

| Model | Modality | | Metric | | |
|---|---|---|---|---|---|
| | Whole-body Radiomics | Non-image | ACC | F1 | AUROC |
| **RF** | | ✓ | **0.578 ± 0.009** | 0.556 ± 0.010 | 0.612 ± 0.003 |
| XGB | | ✓ | 0.565 ± 0.014 | 0.548 ± 0.014 | 0.585 ± 0.010 |
| MLP | | ✓ | 0.558 ± 0.028 | 0.541 ± 0.064 | 0.592 ± 0.020 |
| **RF** | ✓ | | **0.541 ± 0.010** | 0.532 ± 0.021 | 0.548 ± 0.012 |
| XGB | ✓ | | 0.520 ± 0.016 | 0.520 ± 0.014 | 0.524 ± 0.017 |
| MLP | ✓ | | 0.529 ± 0.020 | 0.535 ± 0.031 | 0.536 ± 0.020 |
| **RF** | ✓ | ✓ | **0.545 ± 0.020** | 0.537 ± 0.026 | 0.562 ± 0.009 |
| XGB | ✓ | ✓ | 0.531 ± 0.012 | 0.527 ± 0.020 | 0.544 ± 0.015 |
| MLP | ✓ | ✓ | 0.532 ± 0.021 | 0.557 ± 0.066 | 0.563 ± 0.027 |

**Supplementary Table 1.** Results of tabular models Random Forest (RF), eXtreme Gradient Boosting (XGB), and Mulit-Layer Perceptron (MLP) for cardiovascular disease (CVD) evaluated on the test dataset. Models are trained using different tabular data modalities: whole-body radiomics, non-image data, and a combination of whole-body radiomics and non-image data. The best-performing model based on accuracy is highlighted in bold and reported in Figure 2.

| Model | Modality | | Metric | | |
|---|---|---|---|---|---|
| | Whole-body Radiomics | Non-image | ACC | F1 | AUROC |
| **RF** | | ✓ | **0.627 ± 0.013** | 0.627 ± 0.020 | 0.698 ± 0.010 |
| XGB | | ✓ | 0.597 ± 0.015 | 0.613 ± 0.017 | 0.646 ± 0.005 |
| MLP | | ✓ | 0.608 ± 0.041 | 0.568 ± 0.064 | 0.671 ± 0.041 |
| RF | ✓ | | 0.692 ± 0.026 | 0.695 ± 0.029 | 0.757 ± 0.016 |
| **XGB** | ✓ | | **0.731 ± 0.009** | 0.716 ± 0.011 | 0.785 ± 0.010 |
| MLP | ✓ | | 0.697 ± 0.035 | 0.697 ± 0.051 | 0.763 ± 0.030 |
| RF | ✓ | ✓ | 0.715 ± 0.036 | 0.724 ± 0.036 | 0.772 ± 0.014 |
| **XGB** | ✓ | ✓ | **0.754 ± 0.020** | 0.755 ± 0.022 | 0.798 ± 0.020 |
| MLP | ✓ | ✓ | 0.692 ± 0.010 | 0.709 ± 0.025 | 0.771 ± 0.003 |

**Supplementary Table 2.** Results of tabular models Random Forest (RF), eXtreme Gradient Boosting (XGB), and Multi-Layer Perceptron (MLP) for pancreatic disease evaluated on the test dataset. Models are trained using different tabular data modalities: whole-body radiomics, non-image data, and a combination of whole-body radiomics and non-image data. The best-performing model based on accuracy is highlighted in bold and reported in Figure 2.

| Model | Modality | | Metric | | |
|---|---|---|---|---|---|
| | Whole-body Radiomics | Non-image | ACC | F1 | AUROC |
| **RF** | | ✓ | **0.629 ± 0.022** | 0.627 ± 0.017 | 0.689 ± 0.020 |
| XGB | | ✓ | 0.512 ± 0.015 | 0.662 ± 0.016 | 0.640 ± 0.043 |
| MLP | | ✓ | 0.537 ± 0.071 | 0.555 ± 0.114 | 0.542 ± 0.109 |
| **RF** | ✓ | | **0.624 ± 0.038** | 0.590 ± 0.045 | 0.646 ± 0.014 |
| XGB | ✓ | | 0.595 ± 0.035 | 0.544 ± 0.056 | 0.638 ± 0.020 |
| MLP | ✓ | | 0.588 ± 0.030 | 0.537 ± 0.030 | 0.628 ± 0.038 |
| **RF** | ✓ | ✓ | **0.637 ± 0.034** | 0.606 ± 0.043 | 0.657 ± 0.019 |
| XGB | ✓ | ✓ | 0.600 ± 0.029 | 0.621 ± 0.015 | 0.623 ± 0.021 |
| MLP | ✓ | ✓ | 0.551 ± 0.035 | 0.564 ± 0.065 | 0.594 ± 0.042 |

**Supplementary Table 3.** Results of tabular models Random Forest (RF), eXtreme Gradient Boosting (XGB), and Mulit-Layer Perceptron (MLP) for Liver Disease evaluated on the test dataset. Models are trained using different tabular data modalities: whole-body radiomics, non-image data, and a combination of whole-body radiomics and non-image data. The best-performing model based on accuracy is highlighted in bold and reported in Figure 2.

| Model | Modality | | Metric | | |
|---|---|---|---|---|---|
| | Whole-body Radiomics | Non-image | ACC | F1 | AUROC |
| **RF** | | ✓ | **0.489 ± 0.017** | 0.479 ± 0.015 | 0.483 ± 0.014 |
| XGB | | ✓ | 0.483 ± 0.014 | 0.477 ± 0.022 | 0.478 ± 0.019 |
| MLP | | ✓ | 0.487 ± 0.025 | 0.507 ± 0.062 | 0.495 ± 0.014 |
| RF | ✓ | | 0.590 ± 0.020 | 0.581 ± 0.028 | 0.609 ± 0.018 |
| **XGB** | ✓ | | **0.614 ± 0.008** | 0.602 ± 0.007 | 0.618 ± 0.013 |
| MLP | ✓ | | 0.530 ± 0.022 | 0.505 ± 0.042 | 0.529 ± 0.020 |
| RF | ✓ | ✓ | 0.581 ± 0.013 | 0.580 ± 0.015 | 0.605 ± 0.015 |
| **XGB** | ✓ | ✓ | **0.623 ± 0.028** | 0.604 ± 0.042 | 0.629 ± 0.015 |
| MLP | ✓ | ✓ | 0.513 ± 0.029 | 0.505 ± 0.039 | 0.507 ± 0.044 |

**Supplementary Table 4.** Results of tabular models Random Forest (RF), eXtreme Gradient Boosting (XGB), and Mulit-Layer Perceptron (MLP) for cancer evaluated on the test dataset. Models are trained using different tabular data modalities: whole-body radiomics, non-image data, and a combination of whole-body radiomics and non-image data. The best-performing model based on accuracy is highlighted in bold and reported in Figure 2.

| Model | Modality | | Metric | | |
|---|---|---|---|---|---|
| | Whole-body Radiomics | Non-image | ACC | F1 | AUROC |
| **RF** | | ✓ | **0.698 ± 0.022** | 0.725 ± 0.018 | 0.744 ± 0.022 |
| XGB | | ✓ | 0.653 ± 0.035 | 0.686 ± 0.041 | 0.730 ± 0.010 |
| MLP | | ✓ | 0.657 ± 0.038 | 0.660 ± 0.036 | 0.687 ± 0.038 |
| **RF** | ✓ | | **0.685 ± 0.017** | 0.677 ± 0.023 | 0.751 ± 0.014 |
| XGB | ✓ | | 0.654 ± 0.029 | 0.640 ± 0.046 | 0.734 ± 0.021 |
| MLP | ✓ | | 0.651 ± 0.071 | 0.651 ± 0.058 | 0.703 ± 0.052 |
| **RF** | ✓ | ✓ | **0.743 ± 0.017** | 0.752 ± 0.018 | 0.774 ± 0.022 |
| XGB | ✓ | ✓ | 0.690 ± 0.012 | 0.708 ± 0.019 | 0.767 ± 0.017 |
| MLP | ✓ | ✓ | 0.631 ± 0.035 | 0.647 ± 0.031 | 0.699 ± 0.024 |

**Supplementary Table 5.** Results of tabular models Random Forest (RF), eXtreme Gradient Boosting (XGB), and Multi-Layer Perceptron (MLP) for chronic obstructive pulmonary disease (COPD) evaluated on the test dataset. Models are trained using different tabular data modalities: whole-body radiomics, non-image data, and a combination of whole-body radiomics and non-image data. The best-performing model based on accuracy is highlighted in bold and reported in Figure 2.

| Model | Modality | | Metric | | |
|---|---|---|---|---|---|
| | Whole-body Radiomics | Non-image | ACC | F1 | AUROC |
| RF | | ✓ | 0.580 ± 0.032 | 0.579 ± 0.037 | 0.601 ± 0.024 |
| **XGB** | | ✓ | **0.595 ± 0.015** | 0.595 ± 0.015 | 0.632 ± 0.004 |
| MLP | | ✓ | 0.584 ± 0.021 | 0.622 ± 0.015 | 0.600 ± 0.022 |
| RF | ✓ | | 0.585 ± 0.025 | 0.555 ± 0.029 | 0.643 ± 0.012 |
| **XGB** | ✓ | | **0.623 ± 0.015** | 0.608 ± 0.011 | 0.635 ± 0.014 |
| MLP | ✓ | | 0.597 ± 0.020 | 0.597 ± 0.020 | 0.645 ± 0.012 |
| RF | ✓ | ✓ | 0.598 ± 0.013 | 0.579 ± 0.020 | 0.649 ± 0.012 |
| **XGB** | ✓ | ✓ | **0.636 ± 0.019** | 0.612 ± 0.024 | 0.648 ± 0.025 |
| MLP | ✓ | ✓ | 0.636 ± 0.035 | 0.648 ± 0.049 | 0.674 ± 0.028 |

**Supplementary Table 6.** Results of tabular models Random Forest (RF), eXtreme Gradient Boosting (XGB), and Multi-Layer Perceptron (MLP) for chronic kidney disease (CKD) evaluated on the test dataset. Models are trained using different tabular data modalities: whole-body radiomics, non-image data, and a combination of whole-body radiomics and non-image data. The best-performing model based on accuracy is highlighted in bold and reported in Figure 2.

| Model | Modality | | Metric | | |
|---|---|---|---|---|---|
| | Whole-body Radiomics | Non-image | ACC | F1 | AUROC |
| **RF** | | ✓ | **0.560 ± 0.017** | 0.538 ± 0.010 | 0.588 ± 0.012 |
| XGB | | ✓ | 0.534 ± 0.007 | 0.483 ± 0.021 | 0.578 ± 0.012 |
| MLP | | ✓ | 0.520 ± 0.017 | 0.493 ± 0.080 | 0.544 ± 0.026 |
| **RF** | ✓ | | **0.579 ± 0.018** | 0.546 ± 0.022 | 0.597 ± 0.012 |
| XGB | ✓ | | 0.574 ± 0.009 | 0.560 ± 0.013 | 0.588 ± 0.012 |
| MLP | ✓ | | 0.550 ± 0.014 | 0.544 ± 0.037 | 0.561 ± 0.012 |
| **RF** | ✓ | ✓ | **0.580 ± 0.019** | 0.568 ± 0.020 | 0.606 ± 0.011 |
| XGB | ✓ | ✓ | 0.555 ± 0.023 | 0.541 ± 0.029 | 0.591 ± 0.021 |
| MLP | ✓ | ✓ | 0.541 ± 0.034 | 0.548 ± 0.058 | 0.559 ± 0.033 |

**Supplementary Table 7.** Results of tabular models Random Forest (RF), eXtreme Gradient Boosting (XGB), and Multi-Layer Perceptron (MLP) for osteoarthritis evaluated on the test dataset. Models are trained using different tabular data modalities: whole-body radiomics, non-image data, and a combination of whole-body radiomics and non-image data. The best-performing model based on accuracy is highlighted in bold and reported in Figure 2.

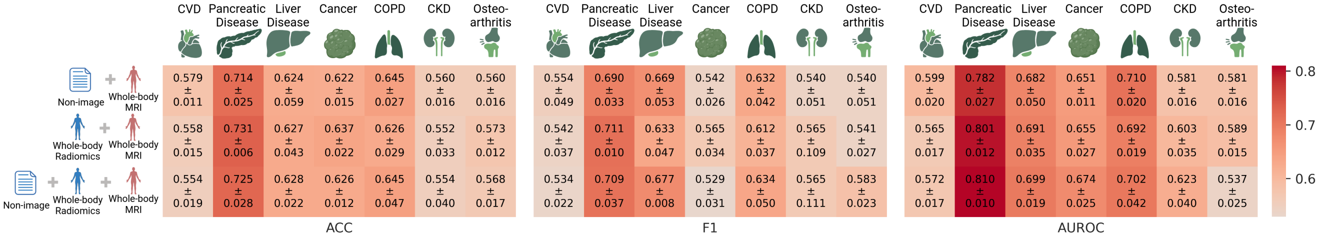


**Supplementary Figure 3.** Results of the best-performing fusion strategy combining whole-body MRI with non-image and whole-body radiomics for 3-year preclinical risk assessment for cardiovascular disease (CVD), pancreatic disease, liver disease, cancer, chronic obstructive pulmonary disease (COPD), chronic kidney disease (CKD), osteoarthritis. Created in BioRender. Seletkov, D. (2025) https://BioRender.com/8hetu2d.

| Modality | Fusion Type | Metric | | |
|---|---|---|---|---|
| | | ACC | F1 | AUROC |
| Non-image + Whole-body MRI | Joint | 0.532 ± 0.009 | 0.546 ± 0.054 | 0.549 ± 0.014 |
| Non-image + Whole-body MRI | **Late** | **0.579 ± 0.011** | 0.554 ± 0.049 | 0.599 ± 0.020 |
| Whole-body Radiomics + Whole-body MRI | Joint | 0.536 ± 0.011 | 0.578 ± 0.033 | 0.562 ± 0.014 |
| Whole-body Radiomics + Whole-body MRI | **Late** | **0.558 ± 0.015** | 0.542 ± 0.037 | 0.565 ± 0.017 |
| Non-image + Whole-body Radiomics + Whole-body MRI | Joint | 0.518 ± 0.024 | 0.570 ± 0.067 | 0.536 ± 0.016 |
| Non-image + Whole-body Radiomics + Whole-body MRI | **Late** | **0.554 ± 0.019** | 0.534 ± 0.022 | 0.572 ± 0.017 |

**Supplementary Table 8.** Late and joint fusion strategies for cardiovascular disease (CVD). The best-performing model based on accuracy is highlighted in bold and reported in Figure 2. Late fusion uses the best-performing model for tabular data, shown in Supplementary Table 1, and the corresponding pre-trained image model for image data.

| Modality | Fusion Type | Metric | | |
|---|---|---|---|---|
| | | ACC | F1 | AUROC |
| Non-image + Whole-body MRI | **Joint** | **0.714 ± 0.025** | 0.690 ± 0.033 | 0.782 ± 0.027 |
| Non-image + Whole-body MRI | Late | 0.708 ± 0.027 | 0.678 ± 0.036 | 0.780 ± 0.014 |
| Whole-body Radiomics + Whole-body MRI | Joint | 0.716 ± 0.007 | 0.699 ± 0.014 | 0.775 ± 0.017 |
| Whole-body Radiomics + Whole-body MRI | **Late** | **0.731 ± 0.006** | 0.711 ± 0.010 | 0.801 ± 0.012 |
| Non-image + Whole-body Radiomics + Whole-body MRI | Joint | 0.705 ± 0.022 | 0.682 ± 0.026 | 0.762 ± 0.025 |
| Non-image + Whole-body Radiomics + Whole-body MRI | **Late** | **0.725 ± 0.028** | 0.709 ± 0.037 | 0.810 ± 0.010 |

**Supplementary Table 9.** Late and joint fusion strategies for pancreatic disease. The best-performing model based on accuracy is highlighted in bold and reported in Figure 2. Late fusion uses the best-performing model for tabular data, shown in Supplementary Table 2, and the corresponding pre-trained image model for image data.

| Modality | Fusion Type | Metric | | |
|---|---|---|---|---|
| | | ACC | F1 | AUROC |
| Non-image + Whole-body MRI | **Joint** | **0.624 ± 0.059** | 0.669 ± 0.053 | 0.682 ± 0.050 |
| Non-image + Whole-body MRI | Late | 0.624 ± 0.044 | 0.631 ± 0.046 | 0.699 ± 0.037 |
| Whole-body Radiomics + Whole-body MRI | Joint | 0.612 ± 0.043 | 0.619 ± 0.031 | 0.692 ± 0.033 |
| Whole-body Radiomics + Whole-body MRI | **Late** | **0.627 ± 0.043** | 0.633 ± 0.047 | 0.691 ± 0.035 |
| Non-image + Whole-body Radiomics + Whole-body MRI | **Joint** | **0.628 ± 0.022** | 0.677 ± 0.008 | 0.699 ± 0.019 |
| Non-image + Whole-body Radiomics + Whole-body MRI | Late | 0.627 ± 0.042 | 0.633 ± 0.047 | 0.698 ± 0.029 |

**Supplementary Table 10.** Late and joint fusion strategies for liver disease. The best-performing model based on accuracy is highlighted in bold and reported in Figure 2. Late fusion uses the best-performing model for tabular data, shown in Supplementary Table 3, and the corresponding pre-trained image model for image data.

| Modality | Fusion Type | Metric | | |
|---|---|---|---|---|
| | | ACC | F1 | AUROC |
| Non-image + Whole-body MRI | **Joint** | **0.622 ± 0.015** | 0.542 ± 0.026 | 0.651 ± 0.011 |
| Non-image + Whole-body MRI | Late | 0.618 ± 0.019 | 0.583 ± 0.019 | 0.638 ± 0.008 |
| Whole-body Radiomics + Whole-body MRI | **Joint** | **0.637 ± 0.022** | 0.565 ± 0.034 | 0.655 ± 0.027 |
| Whole-body Radiomics + Whole-body MRI | Late | 0.627 ± 0.010 | 0.594 ± 0.014 | 0.665 ± 0.009 |
| Non-image + Whole-body Radiomics + Whole-body MRI | **Joint** | **0.626 ± 0.012** | 0.529 ± 0.031 | 0.674 ± 0.025 |
| Non-image + Whole-body Radiomics + Whole-body MRI | Late | 0.624 ± 0.007 | 0.585 ± 0.011 | 0.671 ± 0.013 |

**Supplementary Table 11.** Late and joint fusion strategies for cancer. The best-performing model based on accuracy is highlighted in bold and reported in Figure 2. Late fusion uses the best-performing model for tabular data, shown in Supplementary Table 4, and the corresponding pre-trained image model for image data.

| Modality | Fusion Type | Metric | | |
|---|---|---|---|---|
| | | ACC | F1 | AUROC |
| Non-image + Whole-body MRI | Joint | 0.592 ± 0.044 | 0.591 ± 0.047 | 0.625 ± 0.022 |
| Non-image + Whole-body MRI | **Late** | **0.645 ± 0.027** | 0.632 ± 0.042 | 0.710 ± 0.020 |
| Whole-body Radiomics + Whole-body MRI | Joint | 0.574 ± 0.015 | 0.545 ± 0.048 | 0.614 ± 0.025 |
| Whole-body Radiomics + Whole-body MRI | **Late** | **0.641 ± 0.031** | 0.628 ± 0.032 | 0.701 ± 0.023 |
| Non-image + Whole-body Radiomics + Whole-body MRI | Joint | 0.591 ± 0.043 | 0.576 ± 0.028 | 0.614 ± 0.025 |
| Non-image + Whole-body Radiomics + Whole-body MRI | **Late** | **0.645 ± 0.047** | 0.634 ± 0.050 | 0.702 ± 0.042 |

**Supplementary Table 12.** Late and joint fusion strategies for chronic obstructive pulmonary disease (COPD). The best-performing model based on accuracy is highlighted in bold and reported in Figure 2. Late fusion uses the best-performing model for tabular data, shown in Supplementary Table 5, and the corresponding pre-trained image model for image data.

| Modality | Fusion Type | Metric | | |
|---|---|---|---|---|
| | | ACC | F1 | AUROC |
| Non-image + Whole-body MRI | **Joint** | **0.569 ± 0.010** | 0.610 ± 0.026 | 0.584 ± 0.032 |
| Non-image + Whole-body MRI | Late | 0.544 ± 0.040 | 0.563 ± 0.110 | 0.598 ± 0.036 |
| Whole-body Radiomics + Whole-body MRI | **Joint** | **0.577 ± 0.026** | 0.586 ± 0.040 | 0.594 ± 0.025 |
| Whole-body Radiomics + Whole-body MRI | Late | 0.552 ± 0.033 | 0.565 ± 0.109 | 0.603 ± 0.035 |
| Non-image + Whole-body Radiomics + Whole-body MRI | **Joint** | **0.559 ± 0.014** | 0.603 ± 0.028 | 0.587 ± 0.030 |
| Non-image + Whole-body Radiomics + Whole-body MRI | Late | 0.554 ± 0.040 | 0.565 ± 0.111 | 0.623 ± 0.040 |

**Supplementary Table 13.** Late and joint fusion strategies for chronic kidney disease (CKD). The best-performing model based on accuracy is highlighted in bold and reported in Figure 2. Late fusion uses the best-performing model for tabular data, shown in Supplementary Table 6, and the corresponding pre-trained image model for image data.

| Modality | Fusion Type | Metric | | |
|---|---|---|---|---|
| | | ACC | F1 | AUROC |
| Non-image + Whole-body MRI | Joint | 0.522 ± 0.004 | 0.463 ± 0.081 | 0.546 ± 0.005 |
| Non-image + Whole-body MRI | **Late** | **0.560 ± 0.016** | 0.540 ± 0.051 | 0.581 ± 0.016 |
| Whole-body Radiomics + Whole-body MRI | Joint | 0.514 ± 0.012 | 0.552 ± 0.083 | 0.536 ± 0.014 |
| Whole-body Radiomics + Whole-body MRI | **Late** | **0.573 ± 0.012** | 0.541 ± 0.027 | 0.589 ± 0.015 |
| Non-image + Whole-body Radiomics + Whole-body MRI | Joint | 0.511 ± 0.012 | 0.531 ± 0.086 | 0.529 ± 0.019 |
| Non-image + Whole-body Radiomics + Whole-body MRI | **Late** | **0.568 ± 0.017** | 0.583 ± 0.023 | 0.537 ± 0.025 |

**Supplementary Table 14.** Late and joint fusion strategies for chronic osteoarthritis. The best-performing model based on accuracy is highlighted in bold and reported in Figure 2. Late fusion uses the best-performing model for tabular data, shown in Supplementary Table 7, and the corresponding pre-trained image model for image data.

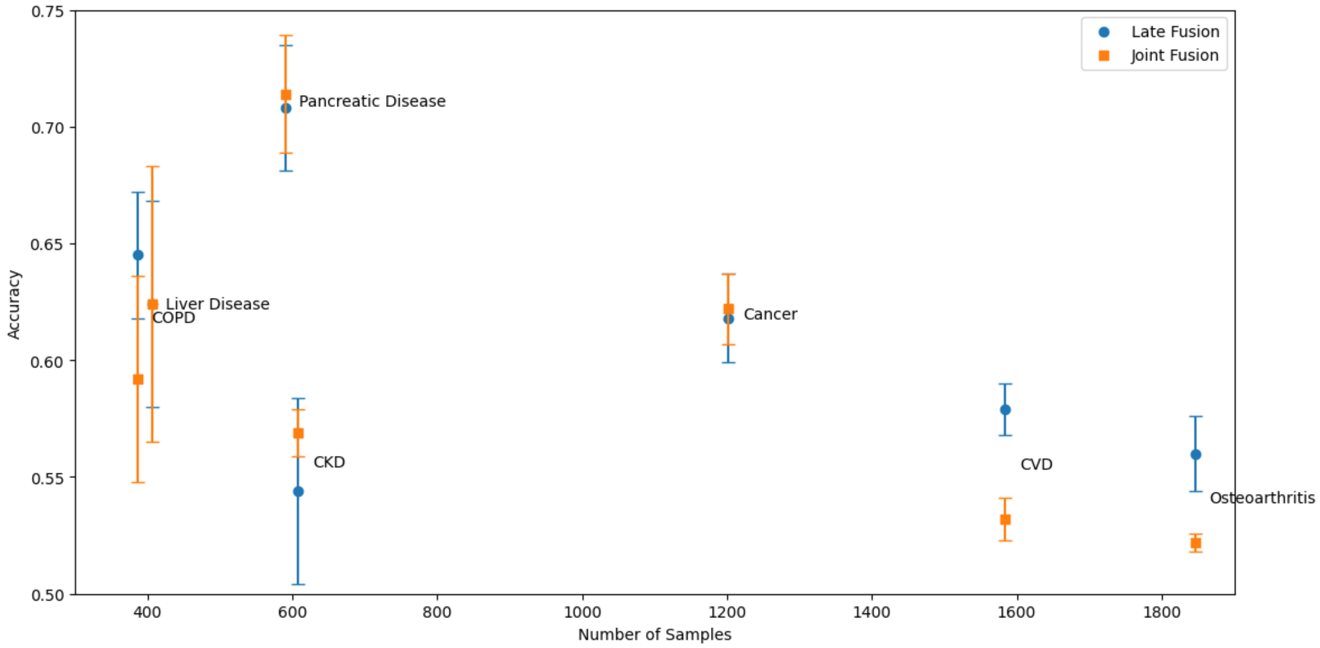


**Supplementary Figure 4.** The mean accuracy of the late and joint fusion strategies compared to the dataset size for "non-image + whole-body MRI" experiments. The results for the construction of this figure are provided in Supplementary Tables 8-14.

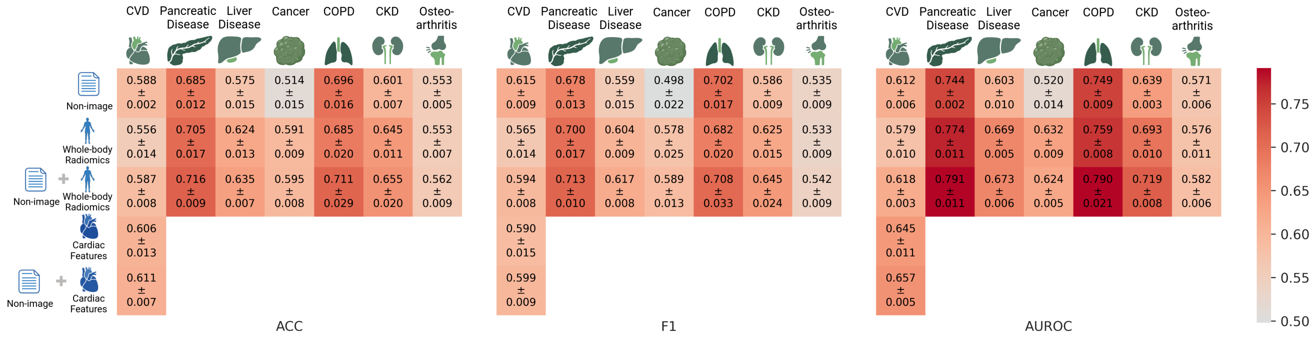


**Supplementary Figure 5.** Results of nested cross-validation of non-image and image-derived tabular models for 3-year preclinical risk assessment for cardiovascular disease (CVD), pancreatic disease, liver disease, cancer, chronic obstructive pulmonary disease (COPD), chronic kidney disease (CKD), osteoarthritis. Image-derived tabular features are represented by whole-body radiomics extracted from whole-body MRI, and cardiac features. Created in BioRender. Seletkov, D. (2025) https://BioRender.com/bmovjrz.

## Supplementary Note 1

Supplementary Figures 6 and 7 present the category feature importances for "non-image + whole-body radiomics" and "whole-body radiomics" experiments, respectively. We apply the model-independent permutation-based feature importance method[2] to the best-performing model by evaluating the decrease in model accuracy when the category features' values are randomly shuffled.

The high importance rank of the non-image category, encompassing all non-image features, aligns with the predictive performance of the non-image data experiment compared to individually evaluated whole-body radiomics categories, shown in Figure 2. Furthermore, the notable importance of the non-image feature category across multiple diseases may be in part explained by its exceptionally rich data matrix, which covers a wide range of disease-related aspects.

The differences between the category feature importances of the "non-image + whole-body radiomics" and "whole-body radiomics" experiments highlight how non-imaging features may already capture information that overlaps with information that is extracted from imaging. For example, the fat category tends to rank lower in the "non-image + whole-body radiomics" experiment, assumably due to the presence of the variables, such as BMI or waist circumference, in the non-image data, as they already provide indirect yet informative proxies for fat distribution. Consequently, the added value of corresponding radiomic features may be reduced when both data sources are combined.

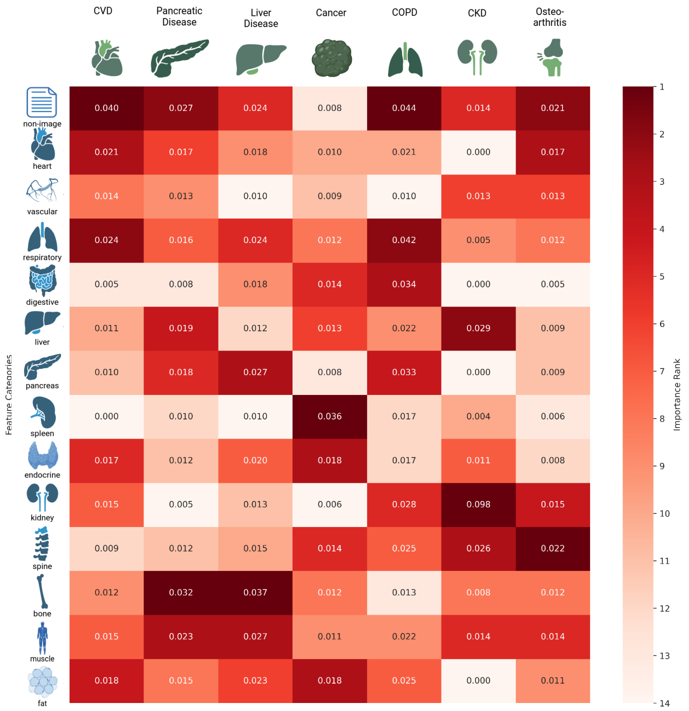


**Supplementary Figure 6.** Mean category-wise feature importances of the best-performing models by the category in the "non-image + whole-body radiomics" experiment. The color represents the rank based on the descending absolute values of the category feature importance. Rank 1 indicates the most important category. Created in BioRender. Seletkov, D. (2025) `https://BioRender.com/200osdb`.

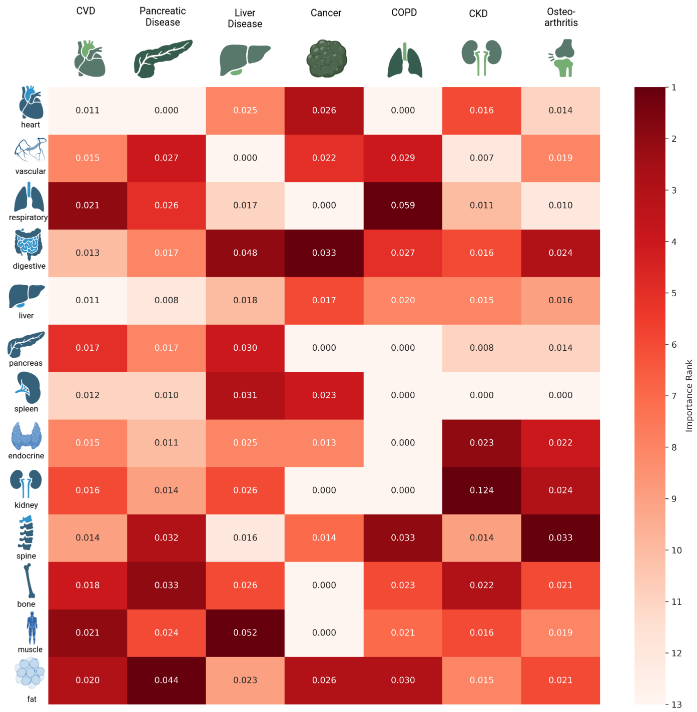


**Supplementary Figure 7.** Mean category-wise feature importances of the best-performing models by the category in the "whole-body radiomics" experiment. The color represents the rank based on the descending absolute values of the category feature importance. Rank 1 indicates the most important category. Created in BioRender. Seletkov, D. (2025) `https://BioRender.com/v3ma0c7`.

| Dataset | Age | BMI | Sex |
|---|---|---|---|
| CVD | 0.904 | **0.000** | 0.879 |
| Pancreatic Disease | 0.846 | **0.000** | 0.666 |
| Liver Disease | 0.776 | **0.000** | 0.921 |
| Cancer | 0.953 | **0.015** | 0.832 |
| COPD | 0.819 | 0.081 | 0.884 |
| CKD | 0.688 | **0.001** | 0.717 |
| Osteoarthritis | 0.922 | **0.000** | 0.779 |

**Supplementary Table 15.** $P$-values for comparison of at-risk and healthy groups, calculated with Welch's t-test for continuous variables age and BMI, and Chi-Squared test for binary variable sex. The statistically significant values ($p$-value $< 0.05$) are highlighted in bold.

| Dataset | Age | BMI | Sex |
|---|---|---|---|
| CVD | 0.006 | 0.242 | 0.008 |
| Pancreatic Disease | 0.016 | 0.698 | 0.036 |
| Liver Disease | 0.029 | 0.430 | 0.010 |
| Cancer | 0.003 | 0.144 | 0.012 |
| COPD | 0.024 | 0.185 | 0.015 |
| CKD | 0.033 | 0.282 | 0.029 |
| Osteoarthritis | 0.005 | 0.212 | 0.013 |

**Supplementary Table 16.** Absolute standardized mean differences ($|\text{SMD}|$) for comparison of at-risk and healthy groups for age, BMI, and sex.

| Model | Hyperparameter Space |
|---|---|
| MLP | hidden_layer_sizes: {[64], [32], [64, 32], [64, 64], [128, 128], [128, 64, 32], {[256, 128, 64], [512, 128], [256, 256], [128, 512]}<br>learning_rate_init: {1e-6, 1e-2}<br>alpha: {0.1, 2.0}<br>batch_size: {64, 256}<br>activation: {relu}<br>early_stopping: {true}<br>validation_fraction: {0.1}<br>n_iter_no_change: {10}<br>solver: {adam}<br>max_iter: {10000} |
| RF | n_estimators: {50, 300}<br>max_depth: {3, 15}<br>min_samples_split: {5, 30}<br>min_samples_leaf: {5, 20}<br>criterion: {gini, entropy, log_loss}<br>max_features: {sqrt, log2}<br>class_weight: {balanced}<br>bootstrap: {true}<br>oob_score: {true} |
| XGB | max_depth: {3, 15}<br>min_child_weight: {3, 10}<br>gamma: {1e-2, 5.0}<br>subsample: {0.6, 0.95}<br>colsample_bytree: {0.6, 0.95}<br>learning_rate: {1e-4, 1e-1}<br>n_estimators: {50, 300}<br>reg_alpha: {1e-2, 5.0}<br>reg_lambda: {1e-2, 5.0}<br>objective: {binary:logistic}<br>eval_metric: {logloss}<br>tree_method: {exact}<br>booster: {gbtree, dart} |

**Supplementary Table 17.** Hyperparameter tuning space for experiments with non-image data and image-derived whole-body radiomics and cardiac features. The investigated models are Multi-layer Perceptron (MLP), Random Forest, eXtreme Gradient Boosting (XGB).

| Modality | Data Augmentation | Optimizer | Scheduler | LR | WD | # Epochs | Dropout |
|---|---|---|---|---|---|---|---|
| Image | Random flips, blur, noise | AdamW | Cosine | 0.001 | 0.0001 | 100 | 0.0 |
| Image + Non-image | Random flips, blur, noise | AdamW | Cosine | 0.0001 | 0.1 | 100 | 0.1 |

**Supplementary Table 18.** Hyperparameters of image (Whole-body MRI) and image + non-image models. LR: Learning Rate, WD: Weight Decay. The experimented model is ResNet 18 3D[3].

## Supplementary Data 1

Supplementary Data 1 contains three tables. The full list of non-image and image-derived tabular features with the corresponding field IDs is provided in Table *Tabular Feature Description*. The full list of the ICD-10 and self-reported codes, alongside fields used to identify the preclinical disease risk assessment datasets, is provided in Table *Diagnostic codes related to disease categories*. The list of segmented organs and the feature categories is provided in Table *Whole-body Radiomics Features and*

*Categories*.

## Supplementary References

[1] Kwan, A. *et al.* Cardiovascular and hepatic disease associations by magnetic resonance imaging: A retrospective cohort study. *Front. Cardiovasc. Medicine* **9**, DOI: 10.3389/fcvm.2022.1009474 (2022).

[2] Breiman, L. Random forests. *Mach. Learn.* **45**, 5–32, DOI: 10.1023/A:1010950718922 (2001).

[3] Tran, D. *et al.* A closer look at spatiotemporal convolutions for action recognition. In *2018 IEEE/CVF Conference on Computer Vision and Pattern Recognition*, 6450–6459, DOI: 10.1109/CVPR.2018.00675 (2018).
